# Solid-state dye-sensitized solar cells based on Zn_1−*x*_Sn_*x*_O nanocomposite photoanodes†

**DOI:** 10.1039/c8ra02852d

**Published:** 2018-07-02

**Authors:** Ayat Nasr El-Shazly, Ahmed Esmail Shalan, Mohamed Mohamed Rashad, Elsayed Ali Abdel-Aal, Ibrahim Ahmed Ibrahim, Mohamed F. El-Shahat

**Affiliations:** Central Metallurgical Research and Development Institute P.O. Box 87, Helwan 11422 Cairo Egypt a.shalan133@gmail.com a.shalan@cmrdi.sci.eg ayatelshazly@gmail.com +202-25010639 +202-25010640-43; Chemistry Department, Faculty of Science, Ain Shams University 11566 Cairo Egypt

## Abstract

Solid-state dye-sensitized solar cells (ss-DSSCs) comprising Sn^2+^-substituted ZnO nanopowder were purposefully tailored *via* a co-precipitation method. The solar cells assembled in this work were sensitized with N719 ruthenium dye and insinuated with spiro-OMeTAD as a solid hole transport layer (HTL). Evidently, significant enhancement in cell efficiency was accomplished with Sn^2+^ ions-substituted ZnO photoelectrodes by maintaining the weight ratio of SnO at 5%. The overall power conversion efficiency was improved from 3.0% for the cell with pure ZnO to 4.3% for the cell with 5% SnO substitution. The improvement in the cell efficiency with Sn^2+^-substituted ZnO photoelectrodes is attributed to the considerably large surface area of the nanopowders for dye adsorption, efficient charge separation and the suppression of charge recombination provided by SnO. Furthermore, the energy distinction between the conduction band edges of SnO and ZnO implied a type II band alignment. Moreover, the durability as well as the stability of 15 assembled cells were studied to show the outstanding long-term stability of the devices made of Sn^2+^ ion substituted ZnO, and the PCE of each cell remained stable and ∼96% of its primary value was retained for up to 100 h. Subsequently, the efficacy was drastically reduced to ∼35% after 250 h of storage.

## Abstract 2

Solid-state dye-sensitized solar cells (ss-DSSCs) comprising Sn^2+^-substituted ZnO nanopowder were purposefully tailored *via* a co-precipitation method.

## Graphical abstract

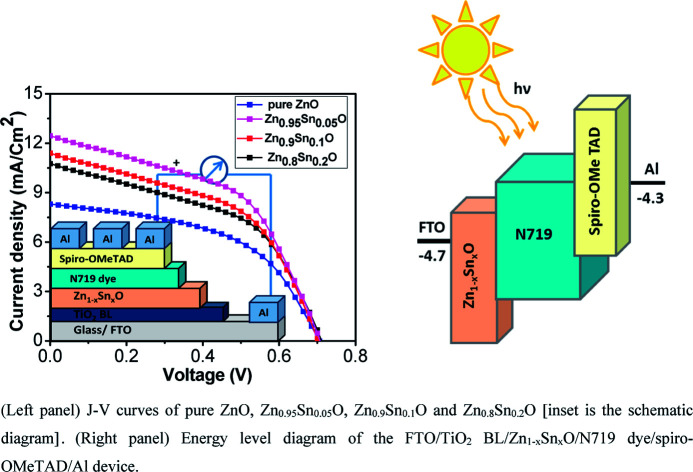

## Introduction

Generally, the most abundant source of renewable energy is solar energy.^1^ To date, single and polycrystalline silicon solar cell technologies have dominated the solar cell market, representing an 80% share.^2^ However, their growth and mass production are restricted because of their high cost, large energy consumption and contamination generated from silicon-based solar cell fabrication. Therefore, many researchers have developed relatively inexpensive and eco-friendly dye-sensitized solar cells (DSSCs) as a substitute for these devices.^3^ In this context, dye sensitized solar cells are the most favorable devices for efficient conversion of light to electricity because of their low production expenditure and simple fabrication.^4–7^ To date, the certified efficiency record for DSSCs is approximately 11.1% for a small cell, and large-scale tests have evidenced the great need for their commercialization.^8^ However, dye-sensitized solar cells established on liquid electrolytes have a precision problem because the redox iodide/tri-iodide (I^−^/I^3−^) solution is corrosive, volatile and photoactive. Furthermore, these cells are heavy, prone to leakage and have complex chemistry.^9,10^ Accordingly, many attempts have been focused on the use of solid-state hole transporting materials (HTMs) to achieve practicability of DSSCs. In this regard, there are many different hole transport materials (conjugated polymers) including pentacene,^11^ poly(triphenyldiamine),^12^ polythiophene,^13^ and poly (3-hexylthiophene) (P3HT),^14^ which can induce charge carrier generation in ss-DSSCs. The most widely utilized hole transfer layer (HTL) is 2,2′,7,7′–tetrakis-(*N*,*N*-di-*p*-methoxyphenylamine)-9,9′-spirobifluorene, also known as a spiro-MeOTAD,^15^ due to its ability to be deposited from solution using different techniques. In 1998, the first solid-state DSSCs were developed by Bach *et al.*;^16^ they described an anatase TiO_2_ dye-sensitized heterojunction with spiro-MeOTAD as an amorphous organic hole-transport material to enhance the photo-induced charge-carrier generation.^17^ Such devices based on ss-DSSCs that integrate solid-state hole-transport materials have recently exhibited power conversion efficiencies of ∼5%.^18^ Moreover, particular interest is currently also concentrated on the alternative metal-oxides including SnO_2,_ SnO, ZnO, Nb_2_O_5_, WO_3_, SrTiO_3_ and Zn_2_SnO_4_.^19,20^ Among these oxides, zinc oxide, ZnO, is a favorable alternatives to TiO_2_ owing to its unique electrochemical features such as large exciton binding energy (60 mV) and high electron mobility (>100 cm^2^ V^−1^ s^−1^), which is higher than that of TiO_2_ (10^−5^ cm^2^ V^−1^ s^−1^),^21^ as well as its low cost, low toxicity and flexible synthesis and morphology. However, the implementation of ZnO photovoltaic cells has been lower than that for TiO_2_-based solar cells, in spite of the reduced recombination rate in the former. This could be due to the lower electron injection efficiency from excited dye molecules into ZnO. Alternatively, SnO has higher electron mobility (131 cm^2^ V^−1^ s^−1^)^22^ and thus, it produces lower number of oxidative holes in the valence band under illumination and also improves the long-term stability of the DSSCs. However, the performance of DSSCs utilizing a SnO photo-anode is worse than that of TiO_2_ because of its 300 mV positive deviation in the conduction band edge compared with TiO_2_ as well as its lower dye regeneration efficiency.^23–25^ In the current study, to overcome the obstacles of individual ZnO and SnO photoanode systems, coupling of the two photoanodes in a nanocomposite structure and controlling the weight ratio of SnO to ZnO is proposed as a possible approach. Some recent reports have focused on nanocomposite photo-anodes and their more efficient electron–hole separation.^26^ Many investigators have exploited different synthetic methods to prepare Zn_1−*x*_Sn_*x*_O nanocomposites including multistep solvent-based routes such as co-precipitation,^27–29^ which is used in this study, hydrothermal^30,31^ and sol–gel.^32,33^ However, to the best of our knowledge, only a few articles have focused on ss-DSSCs with nanocomposite photo-anodes,^34,35^ which reported low efficiencies of up to 0.34 and 0.5% for ss-DSSCs based on ZnO only. Herein, we report the photovoltaic performance of ss-DSSCs solar cells based on Zn_1−*x*_Sn_*x*_O nanocomposites photoanodes as an electron-transport layer prepared *via* the co-precipitation method. The phase composition, crystalline structures, adsorption–desorption isotherm, band gap, and surface area were determined. We observed that the devices based on the Zn_1−*x*_Sn_*x*_O nanocomposites exhibited improved power conversion efficiency and device stability in comparison with those based on pure ZnO.

## Results and discussion

**Fig. 1:**
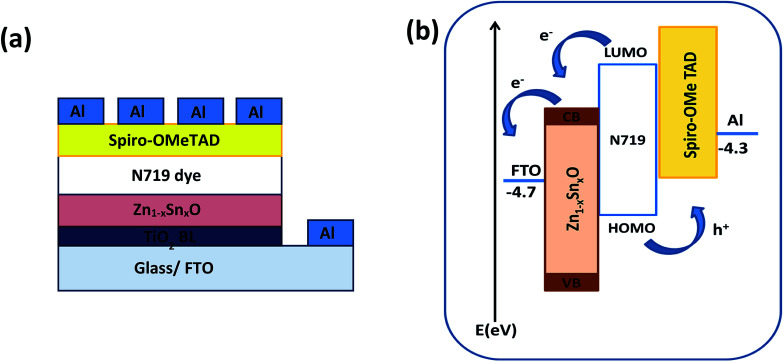
(a) Schematic diagram and (b) energy level diagram of the FTO/TiO_2_ BL/Zn_1−*x*_Sn_*x*_O/N719 dye/spiro-OMeTAD/Al device.

Fig. 1a shows the schematic diagram structure of a device with the configuration glass/FTO/thin TiO_2_ blocking layer/Zn_1−*x*_Sn_*x*_O/N719 dye/spiro-OMeTAD/Al. First, the TiO_2_ blocking layer was coated on a FTO glass substrate through the spray coating technique. The FTO/TiO_2_ blocking layer/Zn_1−*x*_Sn_*x*_O layer was immersed in N719 dye for almost one night, as described elsewhere.^36,37^ Following the formation of the dye layer, a spiro-OMETD HEL was deposited with spin-coating.^38^ The cells' fabrication was completed with the deposition of Al back contact *via* thermal evaporation in a vacuum chamber. Fig. 1b shows the energy levels diagram for each component of the device and the possible charge propagation paths for both photogenerated carriers, electrons (e^−^) and holes (h^+^). In other words, the dissociated charge carriers (e^−^ and h^+^) in the N719 dye layer can be extracted and transferred to the Zn_1−*x*_Sn_*x*_O and spiro-OMETAD layers, respectively, further propagating toward each electrode. The energy level of the conduction band (CB) of Zn_1−*x*_Sn_*x*_O is located below that of the dye, so that a suitable (quasi-)ohmic contact is achieved at the interface.

**Fig. 2:**
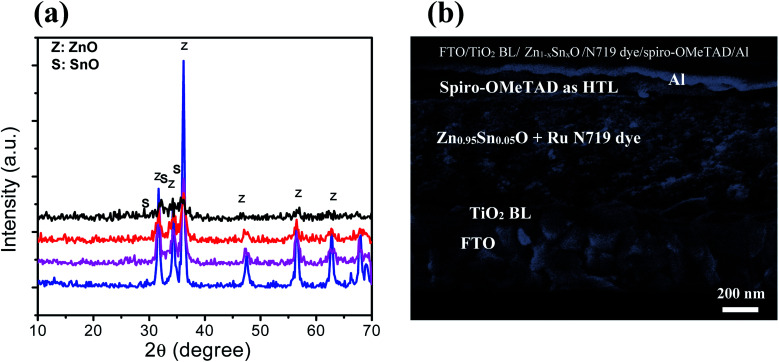
(a) XRD spectra of pure ZnO (blue color), Zn_0.95_Sn_0.05_O (purple color), Zn_0.9_Sn_0.1_O (red color) and Zn_0.8_Sn_0.2_O (black color). (b) Cross sectional view of solid-state DSSCs containing a bi-oxide Zn_0.95_Sn_0.05_O photoelectrode.

Fig. 2a shows the XRD patterns of the ZnO nanoparticles with 0, 5, 10, and 20% weight ratios of SnO, thermally treated at 500 °C for 1 h. The obtained diffraction patterns of the nanopowders suggest that ZnO is crystalline with a hexagonal wurtzite structure. All the XRD patterns showed that no excess impurity phases were present, evidencing that the as-prepared nanopowders were of high purity. The pure ZnO nanoparticles (black color) have a hexagonal structure, as indexed in JCPDS # 89-1397. The substitution of Zn^2+^ with different Sn^2+^ content did not change the hexagonal crystal structure of the wurtzite phase (Fig. 2a; red, purple and blue colors). The diffraction peaks of the 5 wt% SnO-containing nanopowders correspond to the wurtzite ZnO, and there was no SnO peak. With an increase in SnO content to 10 and 20%, peaks from both wurtzite ZnO and tetragonal SnO phases (JCPDS # 01-085-0423) were observed. The crystallinity of the ZnO samples was distorted as the SnO weight ratio was increased, due to degradation in grains as well as crystallite quality. This result can be ascribed to the decrease in the extended to localized state transitions resulting from the band tails formed owing to defects or disorders.^36^ The change in the degree of crystallinity of the composite framework was followed by a moderate change in the absorption edges, as will be discussed while describing the optical properties. Moreover, Fig. 2b clearly depicts the cross section-scanning electron microscopy analysis of the solar cells consisting of N719 Ru dye loaded on the Sn^2+^-substituted ZnO with a 5% weight ratio electrode and infiltrated with spiro-OMeTAD, which exhibited the maximum power conversion efficiency. This confirms the layer by layer formation of the cell. To determine the elemental composition of the as-prepared nanopowders, we performed (EDX) analysis, as illustrated in Fig. S1(a–c) in the ESI†. EDX analysis indicated that the different molar ratios of Zn to Sn were 1 : 0.05, 1 : 0.1 and 1 : 0.2, which were close to the theoretical values. The atomic percentage of tin (Sn) ions in the framework of the formed Zn_1−*x*_Sn_*x*_O nanocomposite was 5, 10 and 20%, as confirmed by the EDX spectrum and indicated in the inset tables of Fig. S1(a–c).†

**Fig. 3:**
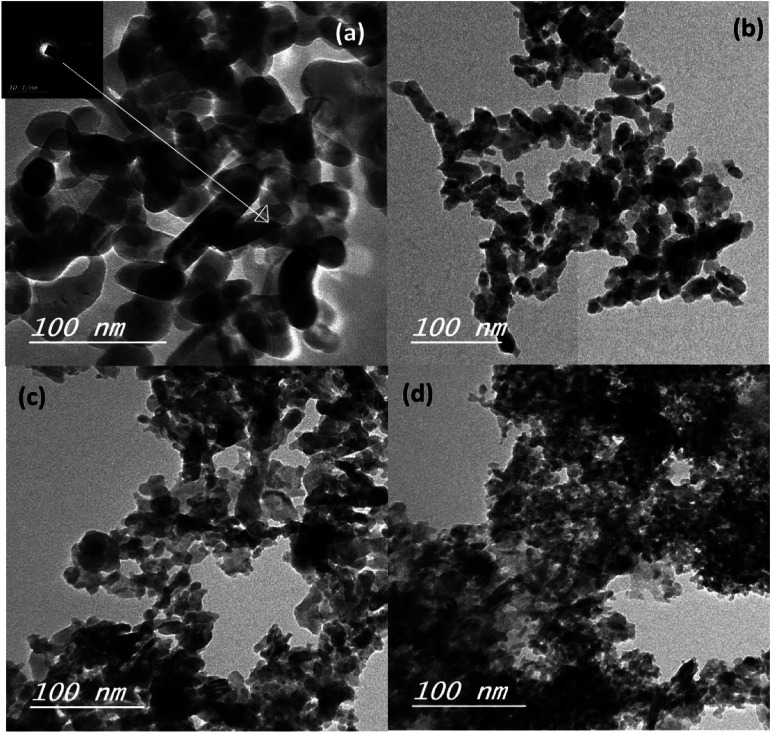
TEM images of (a) the pure ZnO, (b) Zn_0.95_Sn_0.05_O, (c) Zn_0.9_Sn_0.1_O and (d) Zn_0.8_Sn_0.2_O.

The mean particle size of the ZnO–SnO nanocomposites was determined by high resolution transmission electron microscopy (HRTEM), as presented in Fig. 3a–d. Evidently, by comparing the four HRTEM images, we see that the particle size of Sn^2+^-substituted ZnO samples (Fig. 3b–d) are smaller than that of the pure ZnO sample (Fig. 3a). The smaller the particle size of the nanoparticles, the more porous is the generated film, resulting in higher specific surface area and greater amount of dye loading. Furthermore, the inset of Fig. 3a displays the crystallinity of the obtained powders *via* selected area electron diffraction (SAED), which was used to determine the structure of the samples.^36^ Moreover, the obtained results verify the good crystallinity of the pure ZnO sample compared to the Zn_1−*x*_Sn_*x*_O samples. Remarkably, the diameter of the ZnO nanoparticles was ∼25 nm and that of Sn^2+^-substituted ZnO was ∼15 nm, which are in good agreement with the crystallite sizes obtained from the XRD profiles.

**Fig. 4:**
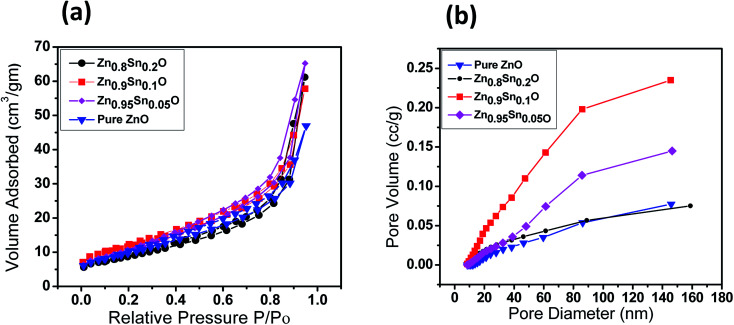
(a) Nitrogen adsorption–desorption isotherms surface area and (b) pore size distribution of pure ZnO, Zn_0.95_Sn_0.05_O, Zn_0.9_Sn_0.1_O and Zn_0.8_Sn_0.2_O.

**Fig. 5:**
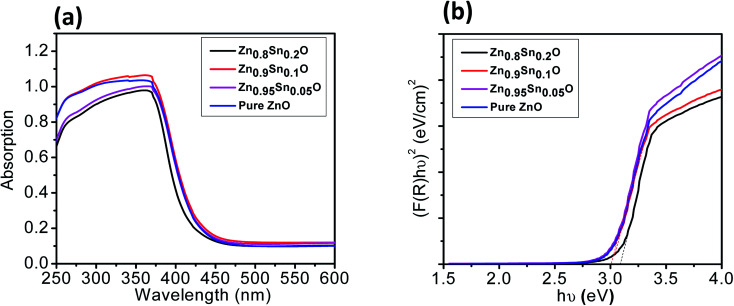
(a) UV-vis and (b) band gap of pure ZnO, Zn_0.95_Sn_0.05_O, Zn_0.9_Sn_0.1_O and Zn_0.8_Sn_0.2_O.

The BET surface area of the as-prepared nanopowders was substantial for the DSSCs due to its impact on the dye loading at the surface of the photoelectrode. A large number of small pores were recognized between the nanoparticles in all samples, suggesting the presence of a mesoporous structure. This phenomenon was assessed by N_2_ sorption analysis. The N_2_ adsorption–desorption isotherm of the pure ZnO and Sn^2+^-substituted ZnO with different Sn content exhibited a type IV isotherm with type H_2_ hysteresis loop (Fig. 4a and b), which is exemplary for mesoporous materials based on the IUPAC classification.^39^ The detailed physical characteristics of the pure ZnO and the Sn^2+^-substituted ZnO samples with different Sn content are given in Table 1. The specific surface area of Sn^2+^-substituted ZnO is relatively high in comparison with that of pure ZnO, which is attributed to the smaller particle sizes of Zn_1−*x*_Sn_*x*_O and is fully consistent with the TEM data.^40^ In order to obtain more evidence for the presence of Sn ions in the formed nanocomposites, we conducted the XPS analysis to determine the chemical components and changes, as shown in Fig. S2(a–c), ESI.† The obtained results illustrate the spectra of the Zn_1−*x*_Sn_*x*_O in the wide 8 range as shown in Fig. S2a† and assigned the existence of (Zn, O and Sn) element peaks. Besides, the narrow scan of ZnO and SnO shows the elemental composition for both of them (Fig. S2b and c†). The Zn 2p_3/2_ peak, (Fig. S2b†) at approximately 1021.6 eV, is assigned to the Zn–O bonds,^25^ which indicates the formation of ZnO composite comprising SnO nanoparticles *via* the solution processable wet chemical method. The XPS spectra of Sn 3d (Fig. S2c†) indicates the presence of spin orbit components, 3d_3/2_ and 3d_5/2_ at binding energies of 486.86 and 493.56 eV, respectively.^25^ These results confirm the existence of Sn^2+^. Notably, Sn ions can enhance the electron transfer between the dye molecules and the photoanodes due to its strong chemical activity through the formation of coordination bonds and oxygen vacancies to enhance the quantity of dye adsorption.^25^Fig. 5a and b depict the absorption spectrum and band gap energy of pure ZnO and Sn^2+^-substituted ZnO with different Sn content. It is well known that ZnO is transparent and has an absorption band centered at around 3.15 eV.^40^ The Tauc model is the easiest tool to calculate the band gaps of Sn^2+^-substituted ZnO with different Sn concentrations.^41^ The optical band gap energy decreased slightly from 3.15 to 3.05 eV when the Sn content was 5%, *i.e.*, the band gap red shifted, but remained constant with the increase in Sn percentage. The insignificant redshift (∼0.1 eV) of the band gap is ascribed to both the compositional change and the structural modification of the composite materials.^36^ Incorporating about 5–20% weight ratio of SnO, as an ionized donor, produces deep states in the band gap that lead to an increase in the scattering of photons by the crystal defects created by the substitution, thus reducing the band gap.^42,43^ More evidence for the formation of Zn_1−*x*_Sn_*x*_O nanocomposites and the positive effect on the cells net efficiency can be obtained by studying dye loading through the absorbance of the dye on each photoanode used in this study, as shown in Fig. S3, ESI.† The results indicate that the amount of dye absorbed by the formed nanocomposite is enhanced compared to the amount that is absorbed by ZnO or SnO nanoparticles alone. This confirms that the charge separation are enhanced and the resistance as well as charge recombination decreases due to the limited penetration of the dye molecules caused by the blocking effect induced by the coated Zn_1−*x*_Sn_*x*_O nanocomposites. The difference in the absorption results clearly indicate and confirm the formation of the nanocomposite.

**Table 1:** Nitrogen sorption–desorption porosimetry studies of pure ZnO and Zn_1−*x*_Sn_*x*_O photoanodes

Sample	*S*_BET_ (m^2^ g^−1^)	Pore volume (cm^3^ g^−1^)	Pore size (nm)
Pure ZnO	34.04	0.36	42.3
Zn_0.95_Sn_0.05_O	49.12	0.35	29.3
Zn_0.9_Sn_0.1_O	61.37	0.41	26.8
Zn_0.8_Sn_0.2_O	65.16	0.39	24.1

**Fig. 6:**
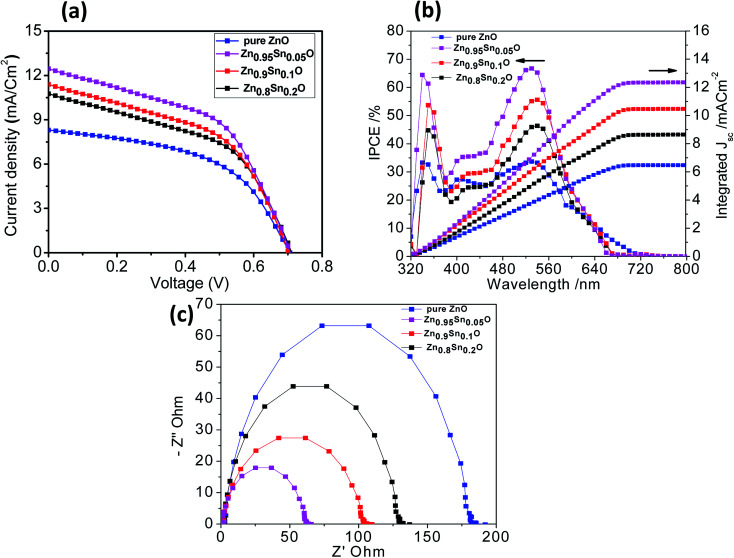
(a) *J*–*V* curves, (b) IPCE spectra and (c) Nyquist diagrams of the impedance spectra of pure ZnO, Zn_0.95_Sn_0.05_O, Zn_0.9_Sn_0.1_O and Zn_0.8_Sn_0.2_O.

Fig. 6a shows the characteristic *J*–*V* curves of the devices fabricated using pure ZnO and Sn^2+^-substituted ZnO with different SnO weight ratios. Furthermore, Table 2 summarizes the photovoltaic parameters for different cells: short circuit photocurrent (*J*_sc_), open-circuit voltages (*V*_oc_), fill factor (FF) and solar conversion efficiency (*η*). It was found that the power conversion efficiency (*η*) enhanced with the addition of SnO, reaching a maximum of 4.3% when the weight ratio of SnO was minimum (5%), with a current density (*J*_SC_) of 12.45 mA cm^−2^ and an open circuit voltage (*V*_oc_) of 0.740 V, which is almost one and half times larger than for pure ZnO. According to the results obtained for the different substituted Zn_1−*x*_Sn_*x*_O samples, there is no significant change in *V*_oc_ upon substitution by SnO due to the fixed difference between the Fermi level of Zn_1−*x*_Sn_*x*_O and the oxidation–reduction potential of HTL. Therefore, the reason for the different performances is mainly due to the different BET surface areas induced by Sn^2+^ substitution. These results can be explained by the electron transfer from the N719 dye into the conduction band (CB) of Zn_1−*x*_Sn_*x*_O. This might be induced due to the coverage of ZnO particles over the SnO particles which favoring the charge injection from the low unoccupied molecular orbital (LUMO) of N719 to the CB of Zn_1−*x*_Sn_*x*_O. Moreover, the photogenerated hole (h^+^) transfer takes place from the high occupied molecular orbital (HOMO) of the N719 dye to the VB of HTL.^44,45^ Increased photocurrent requires good electron transport, large surface area, better dye loading and excellent layer connection strength.^46^ By increasing the weight ratio of SnO to ZnO by 10 and 20%, the efficiency of the cell reduces to 3.9 and 3.4%, respectively. This occurs because the substitution of excess Sn in the ZnO lattice which leads to the formation of a separate SnO phase, resulting in a network of n–n heterojunctions in the ZnO system.^47^ As a result, the free carrier density is decreased, leading to reduce conductivity. Given the parameters of our assembled cells, we can conclude that the difference in fill factor in the tested cells may be related to the difference in shunt and series resistance of the cells. Every cell offers different resistance depending on its structure formation and causes a difference in FF. Moreover, the recombination current in the space charge region of the cell, which is confirmed by the obtained low efficiency, may be one of the reasons behind the difference in FF between the assembled cells. Subsequently, the cell efficiency and the conductivity can be controlled by changing the SnO ratio in the ZnO crystal lattice, which agrees with the results obtained from XRD analysis.^42^

**Table 2:** *I*–*V* characteristics of the ss-DSSCs manufactured using pure ZnO, Zn_0.95_Sn_0.05_O, Zn_0.9_Sn_0.1_O and Zn_0.8_Sn_0.2_O

Samples	*J*_SC_/mA cm^−2^	*V*_OC_/V	FF	*η*/%
ZnO	8.31	0.706	51.10	3.0
Zn_0.95_Sn_0.05_O	12.45	0.740	46.70	4.3
Zn_0.9_Sn_0.1_O	11.40	0.719	47.60	3.9
Zn_0.8_Sn_0.2_O	10.76	0.719	43.90	3.4

Fig. 6b describes the current conversion efficiencies (IPCE) of ZnO and Sn^2+^-substituted ZnO. The ss-DSSC based on the Sn^2+^-substituted ZnO electrode exhibits higher IPCE value over a wide range (from 300 to 700 nm) than pure ZnO. It was reported that IPCE linearly increases with an increase in dye loading. The main functional parameter affecting IPCE is *J*_sc_, which is well correlated with increased dye loading. In fact, typical increase in *J*_sc_ occurs in ss-DSSCs by increasing the optical density of the semiconductor electrode, which enhances the amount of photogenerated charge with a minor effect on *V*_oc._ It can be noticed that the ZnO sample with 5% SnO to ZnO weight ratio exhibits the most significant light harvesting achieved by the dye, compared to that achieved by Sn^2+^-substituted ZnO with 10 or 20% weight ratio and pure ZnO. Thus, we can conclude that the highest IPCE occurred because of the good assembly of dye molecules on the semiconductor electrode, while the small amount of light absorbed by the dye on the pure ZnO electrode is attributed to the deficiency in dye aggregation on the surface of the ZnO electrode.^48^Fig. 6b also shows the integrating photo-current using IPCE results over the AM1.5 solar spectrum at irradiance 100 mW cm^2^. The differences between the current estimated from the IPCE integration and the *J–V* measurements are within the experimental uncertainties. We also note that we tested these cells in air without encapsulation: there is typically a slightly degraded performance for these cells following testing.^17^ To study and obtain better insight into the kinetics and dynamics of the interfacial charge transfer process within the assembled cells, electrochemical impedance spectroscopy (EIS) measurements were performed. Fig. 6c shows the Nyquist plots of the cells based on the different photoanodes used in this study. The results show semicircles for every photoanode that correspond to the electron recombination at the photoelectrode/dye/electrolyte interface. Also, they show that the recombination resistance decreases in the order of pure ZnO > Zn_0.8_Sn_0.2_O > Zn_0.9_Sn_0.1_O > Zn_0.95_Sn_0.05_O photoelectrode prepared using the facile wet chemical method. There is a good relationship between the charge transfer and the net efficiency of the assembled cells. The sample with the higher surface area and faster electron transfer rate had higher cell efficacy and *vice versa*.

**Fig. 7:**
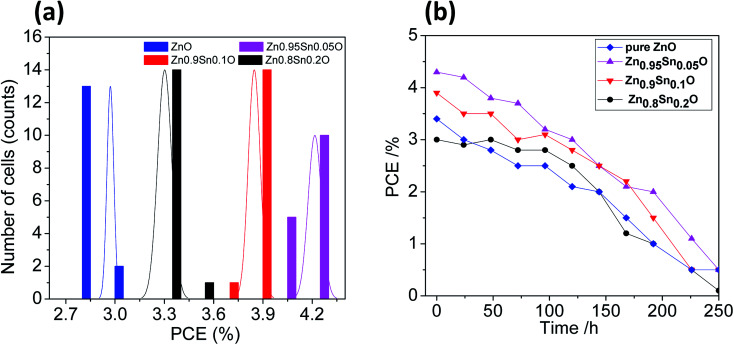
(a) The PCE distribution histogram and (b) the PCE evolution of encapsulated devices stored in ambient air for different time periods [pure ZnO, Zn_0.95_Sn_0.05_O, Zn_0.9_Sn_0.1_O and Zn_0.8_Sn_0.2_O].

The reproducibility of the devices was tested by fabricating 15 identical devices according to the same experimental procedure for each electrode. The corresponding photovoltaic parameters for the ZnO and Sn^2+^-substituted ZnO devices with ratios of 5, 10 and 20% are summarized in Tables S1–S4, in the ESI,† respectively. Fig. 7a shows histograms of the PCE distributions for the devices made with these electrodes for comparison; the corresponding histograms for the other photovoltaic parameters are shown in Fig. S4, ESI.† Our results show that the performances of these devices showed good reproducibility with an average PCE (%) = 2.98 ± 0.02, 4.22 ± 0.04, 3.85 ± 0.04, and 3.30 ± 0.04 for devices made of ZnO and Sn^2+^-substituted ZnO with ratios of 5, 10 and 20%, respectively. To test their long-term stability, we also checked the durability of the devices with different ratios under dark conditions; the corresponding stability results are described in Fig. 7b. Clearly, the cells display good stability over a time period of 250 h. The efficiency of each cell remained stable and 96% of its primary value was retained until 100 h. Subsequently, the efficiency decreased to 35% after 250 h of storage.

## Conclusion

In summary, photoelectrodes of Sn^2+^-substituted ZnO with different Sn content sensitized with N719 and spiro-OMeTAD were utilized to fabricate solid-state DSSCs. Pure ZnO and Sn^2+^-substituted ZnO were prepared by the fast, facile and inexpensive co-precipitation method. The effect of SnO on the properties of ZnO photoelectrodes was investigated. Clearly, the 5% weight ratio SnO-substituted ZnO cell has a high power conversion efficiency compared with that of the pure ZnO solar cell. The short-circuit current (*J*_sc_) and the open circuit voltage (*V*_oc_) increase with the addition of SnO due to the large surface area for dye loading and the better inhibition of electron–hole recombination. Notably, the 4.3% efficiency recorded for the 5% weight ratio Sn^2+^-substituted ZnO cell is favorable for the development of ss-DSSc. Moreover, there is a good relationship between the charge transfer and the net efficiency of the assembled cells. The samples with larger surface area and faster electron transfer rate have higher cell efficacy and *vice versa*. Moreover, fifteen devices with each type of electrode were fabricated to examine the reproducibility of the performance and the stability of the devices, which showed the average PCE of the same order.

## Experimental methods

### Preparation of pure ZnO and Sn^2+^-substituted ZnO nanopowders

Zinc sulphate heptahydrate (ZnSO_4_·7H_2_O) purchased from Dop Organic Chemical was used as a source of Zn^2+^. Moreover, stannous chloride dihydrate (SnCl_2_·2H_2_O) from Dop Organic Chemical was employed as the source of Sn^2+^ through the co-precipitation method. Ammonium hydrogen carbonate (NH_4_HCO_3_) (2 M), (purchased from Riedel-de Haën) was used as a base and diethyl amine was used as a stabilizer to obtain the desired pH value of 11. Sn^2+^-substituted ZnO at different Sn^2+^ molar ratios 0.0, 0.05, 0.1 or 0.2 were prepared through a facile co-precipitation strategy.^36^ Typically, the procedure involved dissolving zinc sulfate (ZnSO_4_) in an aqueous ammonium hydrogen carbonate (NH_4_HCO_3_) solution (2 M), and then adjusting the pH to the desired value of 11 by using diethyl amine as a stabilizer. A milky white precipitate was observed in each case. Then, the precipitate was filtered, washed many times with deionized water and then dried at 60 °C for 24 h. Eventually, the dried precursors of the as-prepared Sn^2+^-substituted ZnO nanopowders at different concentrations were heated in a static air furnace at 500 °C for 1 h.

### Fabrication of electrodes and solid-state DSSC

A 1.0 g portion of ZnO or Sn^2+^-substituted ZnO (tailored using the co-precipitation route) was added to 1.0 mL of deionized water and 5 mL of absolute ethanol, and gently stirred by a hot plate magnetic stirrer for 10 h to form the required paste. The next stages were similar to those described previously.^17^

### Structural and morphological characterization

The phase evolution was distinguished by X-ray diffraction (Brucker axis D8 diffractometer) using Cu-Kα (*λ* = 1.5406) radiation operating at 40 kV and 30 mA at a rate of 2° min^−1^. The microstructures of ZnO and Sn^2+^-substituted ZnO were inspected using transmission electron microscopy (TEM, JEOL 2100) and field emission scanning electron microscopy (FESEM, JEOL JSM-5410) for detecting the cross section of the assembled solar cell along with EDX measurements of the obtained samples. The specific surface area, pore size, pore volume and average pore size were determined using an ASAP 2020 (Micromeritics Instruments, USA) nitrogen adsorption apparatus. X-ray photo-electron spectroscopy (XPS) studies were performed by using a Thermo Scientific K-ALPHA, XPS machine, England. The recorded binding energies were calibrated by taking the C 1s peak at 285.0 eV as a reference.

### Optical and photovoltaic measurements

A UV vis-NIR scanning spectrophotometer (Jasco-V-570 Spectrophotometer, Japan) was employed to obtain UV-vis absorption spectra. Photocurrent–voltage *J*–*V* characteristic measurements were examined using a solar simulator.^37^ The incident monochromatic photoelectric conversion efficiency (IPCE) analyses were performed using a QE/IPCE measurement system from Oriel at 10 nm intervals between 300 and 800 nm, where a monochromator was employed to acquire monochromatic light from a 300 W Xe lamp. Furthermore, electrochemical impedance spectroscopy measurements (EIS) were conducted with a computer-controlled potentiostat (EG&G, M273) equipped with a frequency response analyzer (EG&G, M1025). The frequency range is 0.005–100 kHz. The magnitude of the alternative signal is 10 mV using a 450 W xenon light source.

## Author Contributions

A. N. E. and A. E. S. contributed equally to this study by performing the experiments and writing the manuscript.

## Funding

This study was supported by grants from the Central Metallurgical Research and Development Institute (CMRDI), grant no. 00025.

## Conflicts of interest

The authors declare no competing financial interest.

## Supplementary Material

RA-008-C8RA02852D-s001
