# Expression of Concern: Solid-state dye-sensitized solar cells based on Zn_1−*x*_Sn_*x*_O nanocomposite photoanodes

**DOI:** 10.1039/d3ra90109b

**Published:** 2023-11-08

**Authors:** Ayat Nasr El-Shazly, Ahmed Esmail Shalan, Mohamed Mohamed Rashad, Elsayed Ali Abdel-Aal, Ibrahim Ahmed Ibrahim, Mohamed F. El-Shahat

**Affiliations:** a Central Metallurgical Research and Development Institute P.O. Box 87 Helwan 11422 Cairo Egypt a.shalan133@gmail.com a.shalan@cmrdi.sci.eg ayatelshazly@gmail.com +202-25010639 +202-25010640-43; b Chemistry Department, Faculty of Science, Ain Shams University 11566 Cairo Egypt

## Abstract

Expression of Concern for ‘Solid-state dye-sensitized solar cells based on Zn_1−*x*_Sn_*x*_O nanocomposite photoanodes’ by Ayat Nasr El-Shazly *et al.*, *RSC Adv.*, 2018,**8**, 24059–24067, DOI: https://doi.org/10.1039/c8ra02852d.

The Royal Society of Chemistry is publishing this expression of concern in order to alert readers that concerns have been raised regarding the reliability of the IPCE data in Fig. 6b, the Nyquist data in Fig. 6c, the EDX data in Fig. S1c and the XPS data in Fig. S2. An investigation is underway, and an Expression of Concern will continue to be associated with the article until a final outcome is reached.

Laura Fisher

02/11/2023

Executive Editor, *RSC Advances*

## Supplementary Material

