# Supplementary material for: Solid-state dye-sensitized solar cells based on Zn1−xSnxO nanocomposite photoanodes
Source: RSC Adv. 2018 Jul 2;8(42):24059–67. doi: 10.1039/c8ra02852d (PMC9081753; doi:10.1039/c8ra02852d)
Supplement: RA-008-C8RA02852D-s001 [file RA-008-C8RA02852D-s001.pdf]

Electronic Supplementary Material (ESI) for RSC Advances.
This journal is © The Royal Society of Chemistry 2018

Supporting Information

Solid State Dye-Sensitized Solar Cells Based on $Zn_{1-x}Sn_xO$ Nanocomposite Photoanodes

Ayat Nasr El-Shazly[1†], Ahmed Esmail Shalan[1*†], Mohamed Mohamed Rashad[1], Elsayed Ali Abdel-Aal[1], Ibrahim Ahmed Ibrahim[1] and Mohamed F. El-Shahat[2]*

[1] Central Metallurgical Research and Development Institute, P.O. Box 87, Postal code: 11422, Helwan, Cairo, Egypt
[2] Chemistry Department, Faculty of Science, Ain Shams University, Postal code: 11566, Cairo, Egypt

[*] Corresponding Author: E-mail: (A. E. Shalan) a.shalan133@gmail.com; a.shalan@cmrdi.sci.eg
Fax: +202-25010639; Tel: +202-25010640-43, 11422, Cairo, Egypt.
E-mail: (A. N. El-Shazly) ayatelshazly@gmail.com; Fax: +202-25010639; Tel: +202-25010640-43, 11422, Cairo, Egypt.
† (A. N. El-Shazly) and (A. E. Shalan) contribute equally to this manuscript.

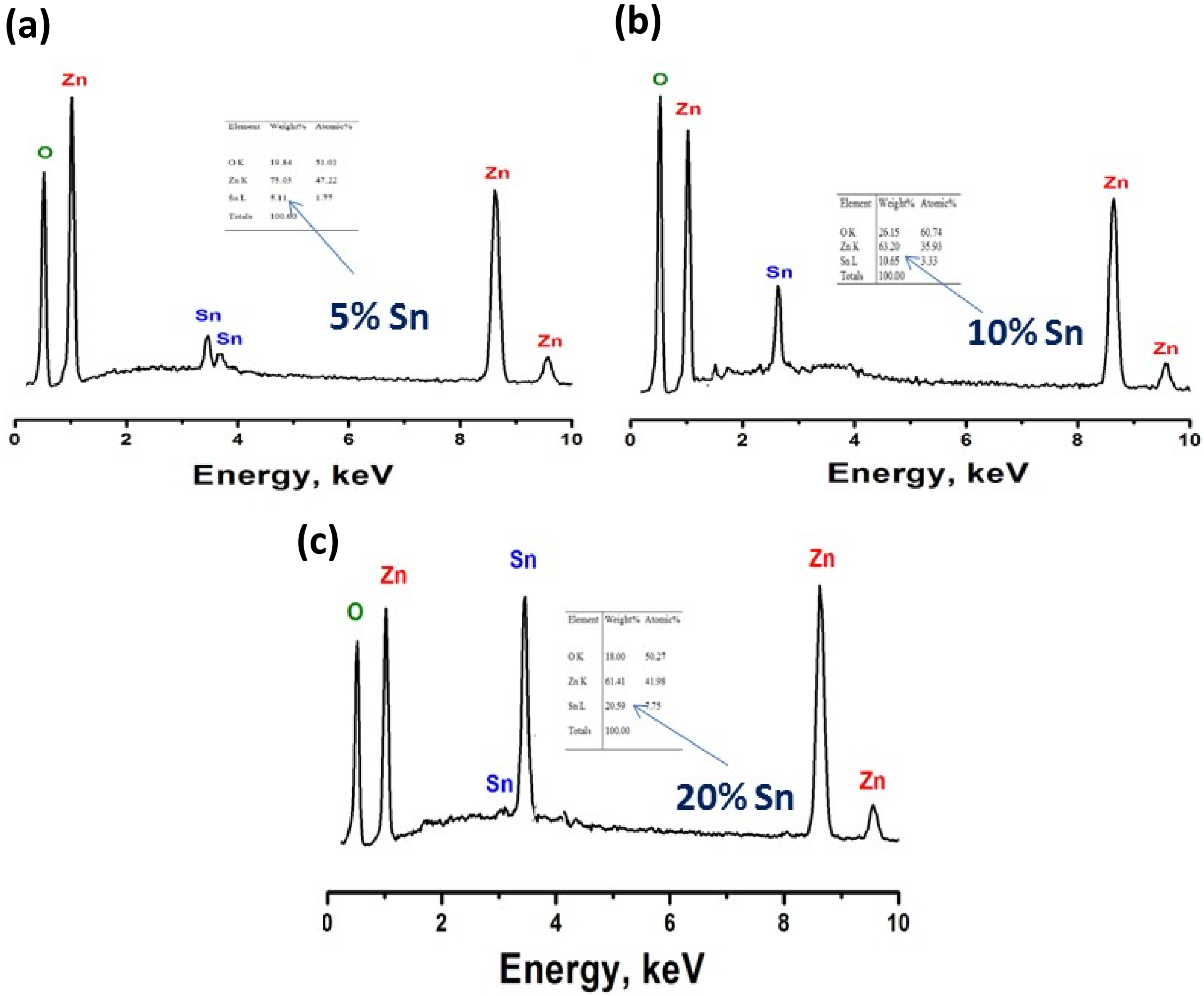

Figure S1: Energy dispersive X-ray spectroscopy (EDX) analysis data of the produced nanocomposites

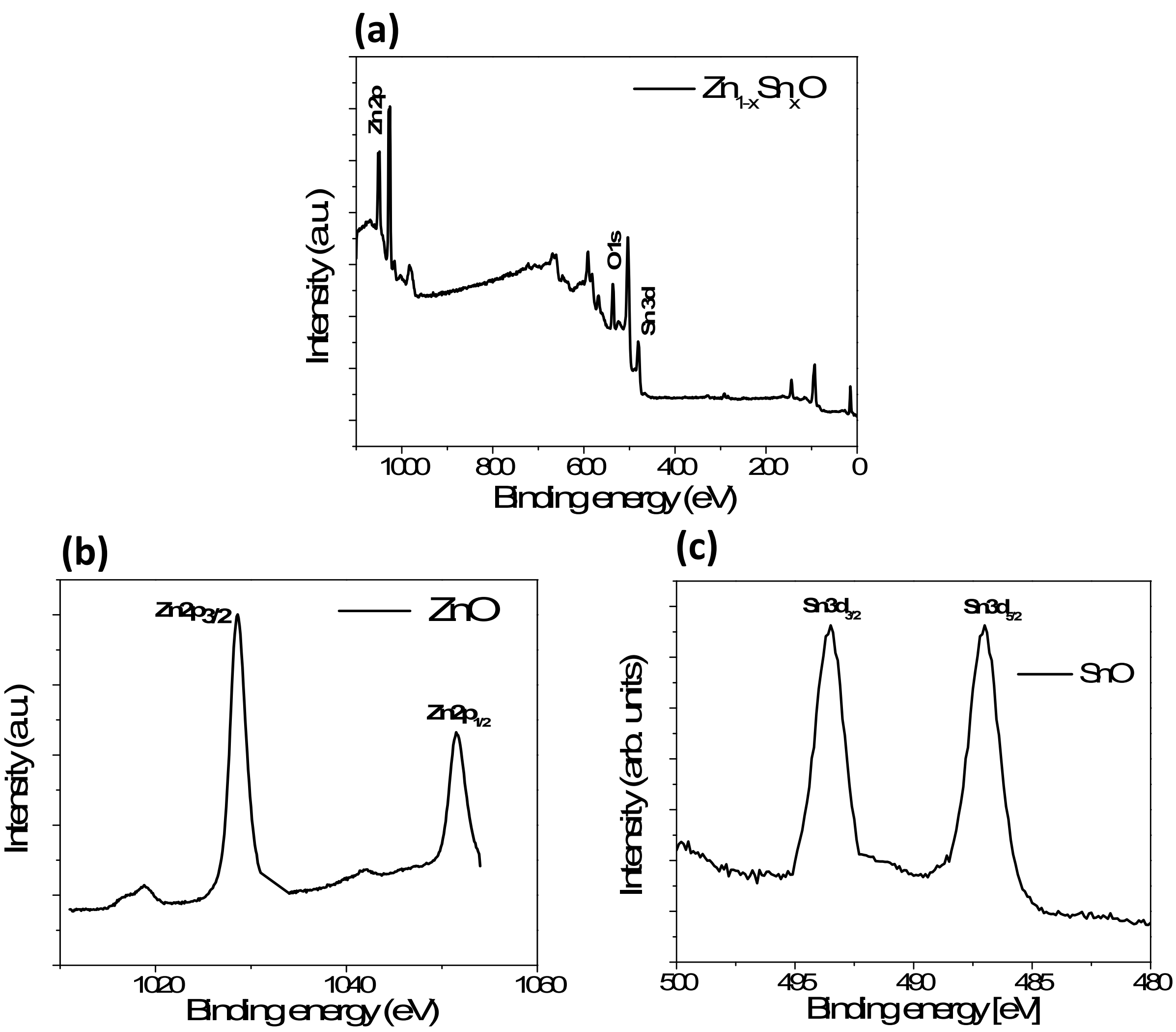

Figure S2: XPS of (a) wide range of $Zn_{1-x}Sn_xO$ nanocomposites, (b) and (c) narrow range of SnO and ZnO nanoparticles, respectively

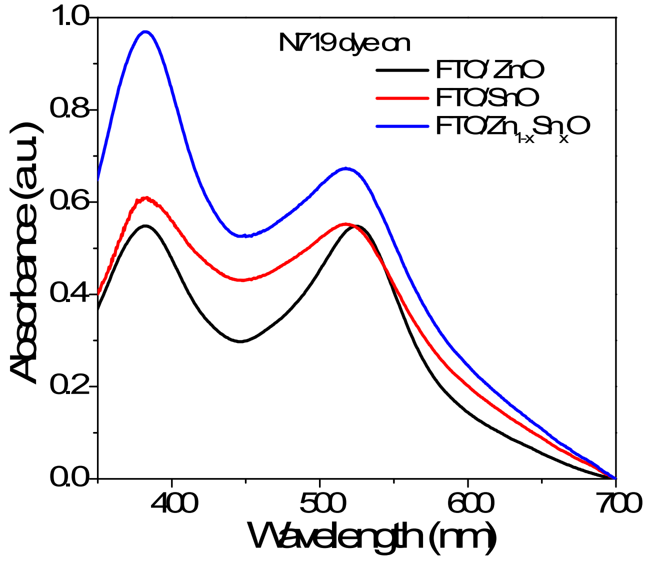

Figure S3: Optical absorbance of the dissolved-out dyes of the pure SnO, the pure ZnO and $Zn_{1-x}Sn_xO$ nanocomposite, respectively loaded on Ru N719 dye.

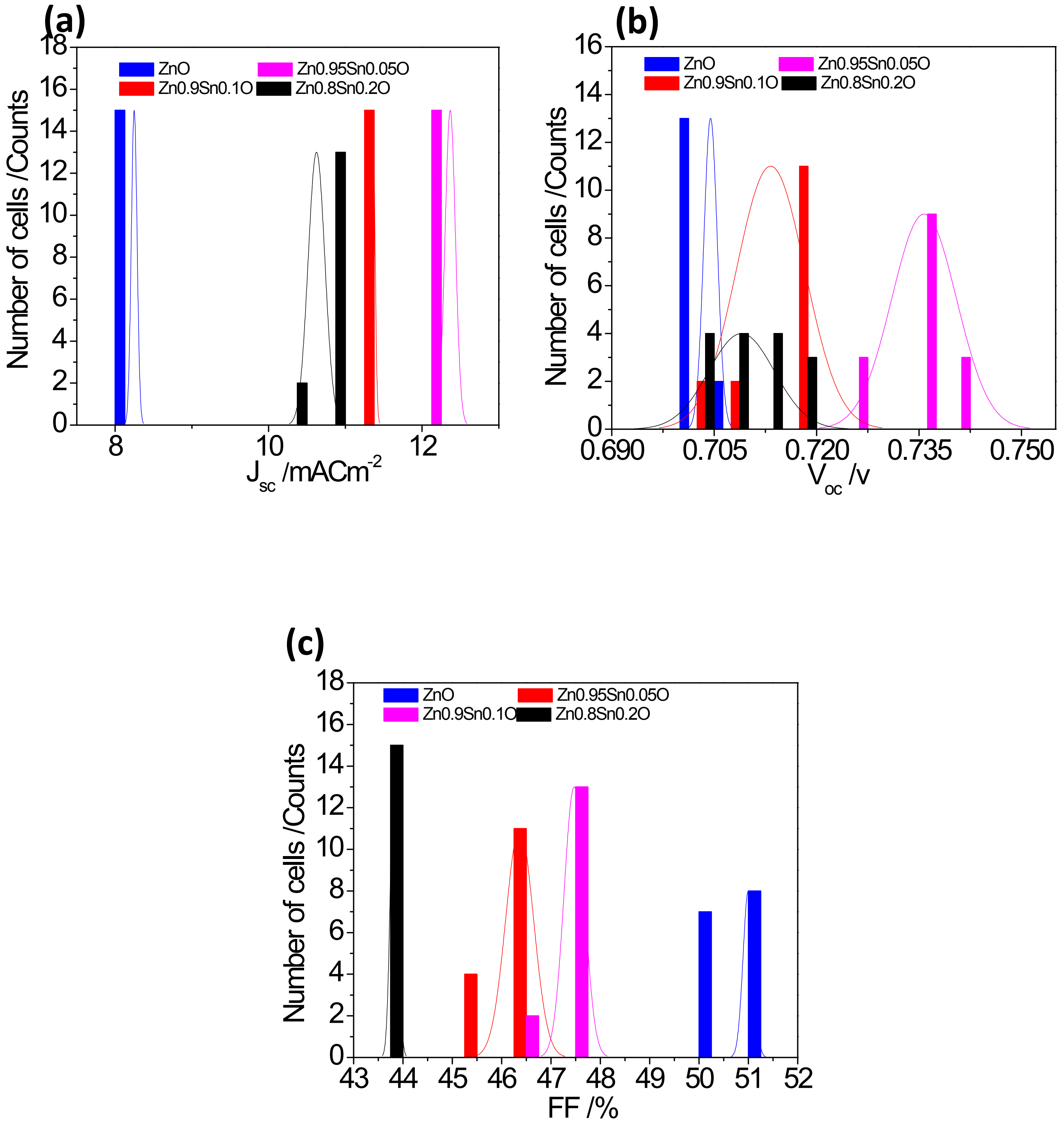

Figure S4. The J_{sc}, V_{oc} and FF distribution histogram (a, b and c), respectively of the pure ZnO (blue color), $Zn_{0.95}Sn_{0.05}O$ (purple color), $Zn_{0.9}Sn_{0.1}O$ (red color) and $Zn_{0.8}Sn_{0.2}O$ (black color), respectively.

Table S1. Photovoltaic parameters of fifteen ss-DSSCs devices made of pure ZnO under simulated AM-1.5G illumination (power density 100 mW cm^{-2}) with active area 0.25 cm^2.

# of cells	J_{sc}(mA/cm^2)	V_{oc} (V)	FF (%)	PCE (%)
1	8.31	0.706	51.10	3.00
2	8.30	0.705	51.00	2.98
3	8.25	0.705	50.99	2.97
4	8.26	0.704	50.99	2.97
5	8.26	0.705	50.98	3.00
6	8.25	0.706	50.98	2.97
7	8.27	0.704	51.11	2.98
8	8.21	0.705	51.10	2.95
9	8.22	0.703	51.00	2.99
10	8.23	0.705	51.00	2.96
11	8.30	0.703	51.10	2.98
12	8.19	0.704	50.90	2.93
13	8.19	0.704	50.95	2.94
14	8.24	0.703	51.09	2.96
15	8.24	0.705	50.69	2.99
**average**	8.25±0.03	0.704±0.001	50.99±0.10	2.98±0.02

Table S2. Photovoltaic parameters of fifteen ss-DSSCs devices made of $Zn_{0.95}Sn_{0.05}O$ under simulated AM-1.5G illumination (power density 100 mW cm^{-2}) with active area 0.25 cm^2.

# of cells	J_{sc}(mA/cm^2)	V_{oc} (V)	FF (%)	PCE (%)
1	12.45	0.740	46.70	4.30
2	12.40	0.735	46.50	4.23
3	12.35	0.735	46.56	4.22
4	12.45	0.740	46.60	4.29
5	12.40	0.736	45.99	4.19
6	12.38	0.739	46.25	4.23
7	12.39	0.736	46.67	4.26
8	12.40	0.725	46.62	4.19
9	12.43	0.729	46.57	4.21
10	12.36	0.728	46.37	4.17
11	12.35	0.737	45.95	4.18
12	12.25	0.739	45.93	4.16
13	12.25	0.738	46.45	4.20
14	12.25	0.739	46.34	4.20
15	12.39	0.740	45.96	4.21
**average**	12.36±0.06	0.736±0.001	46.36±0.28	4.22±0.04

Table S3. Photovoltaic parameters of fifteen ss-DSSCs devices made of $Zn_{0.9}Sn_{0.1}O$ under simulated AM-1.5G illumination (power density 100 mW cm^{-2}) with active area 0.25 cm^2.

# of cells	J_{sc}(mA/cm^2)	V_{oc} (V)	FF (%)	PCE (%)
1	11.40	0.719	47.60	3.90
2	11.35	0.715	47.55	3.86
3	11.40	0.716	47.56	3.88
4	11.30	0.715	47.50	3.84
5	11.33	0.705	47.55	3.81
6	11.34	0.706	47.58	3.81
7	11.36	0.705	47.58	3.81
8	11.38	0.706	46.99	3.78
9	11.33	0.715	46.95	3.80
10	11.34	0.716	47.48	3.86
11	11.36	0.715	47.59	3.87
12	11.38	0.718	47.52	3.88
13	11.38	0.718	47.50	3.88
14	11.35	0.715	47.55	3.86
15	11.37	0.715	47.55	3.87
**average**	11.36±0.03	0.713±0.0005	47.47±0.21	3.85±0.04

Table S4. Photovoltaic parameters of fifteen ss-DSSCs devices made of $Zn_{0.8}Sn_{0.2}O$ under simulated AM-1.5G illumination (power density 100 mW cm^{-2}) with active area 0.25 cm^2.

# of cells	J_{sc}(mA/cm^2)	V_{oc} (V)	FF (%)	PCE (%)
1	10.76	0.719	43.90	3.40
2	10.75	0.705	43.85	3.32
3	10.74	0.705	43.88	3.32
4	10.75	0.703	43.87	3.32
5	10.75	0.715	43.88	3.37
6	10.68	0.706	43.85	3.31
7	10.50	0.706	43.85	3.25
8	10.55	0.706	43.89	3.27
9	10.58	0.703	43.90	3.27
10	10.48	0.708	43.85	3.25
11	10.46	0.710	43.75	3.25
12	10.59	0.710	43.70	3.29
13	10.53	0.715	43.68	3.29
14	10.58	0.712	43.75	3.30
15	10.66	0.712	43.76	3.32
**average**	10.62±0.11	0.709±0.005	43.82±0.07	3.30±0.04
